# The Relationship Between Screen and Outdoor Time With Rates of Myopia in Spanish Children

**DOI:** 10.3389/fpubh.2020.560378

**Published:** 2020-10-14

**Authors:** Cristina Alvarez-Peregrina, Miguel Ángel Sánchez-Tena, Clara Martinez-Perez, Cesar Villa-Collar

**Affiliations:** School of Biomedical and Health Science, Universidad Europea de Madrid, Madrid, Spain

**Keywords:** smartphone, outdoors-time, myopia, vision, children, prevention, screen- time, device

## Abstract

**Background:** Nowadays, digital devices have become usual in children's lives around the world. Five percent of the children between 5 and 7 years old have their own smartphone and forty-two percent of them have their own tablet. This fact has produced a change in their lifestyle that can imply some risks, threats and/or opportunities. The light emitted by digital devices' screens could involve, among others, possible risks to children's vision.

**Methods:** This study shows a detailed analysis of the vision of 7,497 children between 5 and 7 years old carried out in the “Annual school campaign for children's visual health” in Spain during the years 2016, 2017, and 2019. The study connects the results in the visual screening with children's lifestyle, taking into account both, the number of hours per day that they use all digital devices and the daily time of outdoor exposure.

**Results:** The study shows that children with myopia have more screen time use and shorter outdoor activity time when compared to those without myopia (*p* < 0.01).

**Conclusions:** Myopia in children is a public health problem and requires healthy lifestyle interventions at individual as well as at community level.

## Introduction

Children's behavior has changed across different generations (baby boomers, millennials, generation Z, generation T, etc.). Children born since 2010 belong to generation T and know the surrounding world through a digital screen, having a behavior conditioning by immediacy, hypoconnectivity, and speed (1–4). Thus, the children of today grow up surrounded by devices such as smartphones, televisions, tablets or computers, which have become a fundamental part of their daily lives, using these electronic devices as well as at school as at home (2–4).

The continued use of electronic devices has had a direct influence on the competences and interests of these children, and likewise, their way of learning is completely different from that of previous generations. Prensky already defined in 2001 the expression “Digital Natives” as people that had “spent their entire lives surrounded by and using computers, videogames, digital music players, video cams, cell phones, and all the other toys and tools of the digital age” (1). Broadly speaking, digital natives prefer experiential learning, are used to multitasking and tend to be completely dependent on communication technology for access to information and their interaction with others (1, 5–8).

However, the increasing use of this technology and the continued exposure of children to the screens of different devices also brings with it significant risks. It is important to take into consideration the duration, content, nighttime use and the number of digital devices when determining the effect that screen time has on physical and mental health (9). There is evidence of an association between screen time and a higher risk of adiposity, dietary problems, symptoms of depression and a lower quality of life among children and adolescents (10, 11).

Screen time increases every year. In 2018, 5% of children aged 5 to 7 years worldwide already owned a mobile phone and 42% had their own tablet (12). Furthermore, the average screen time use in children aged between 8 and 12 has been increasing 49 min a day over the last 3 years with an average screen time per day of 4 h and 18 min in 2016 up to 5 h and 7 min in 2019 (13). This increase in screen time has visual implications. In this way, there is a growing concern about the risks that both, the increase in screen time and the reduction of time spent outdoors by children as a result of this changed lifestyle, could pose. In particular, there is concern about children developing myopia, which in turn may affect their academic achievements.

Myopia is currently considered as one of the main public health problems worldwide (14). The prevalence of myopia varies geographically; in Asia, the percentage of school-age children with myopia reaches 80–90% in various regions (15). These values are lower in Europe, with a prevalence of 20% among Spanish children aged between 5 and 7 years (16). As mentioned above, recent studies have found that spending more time outdoors attenuates the appearance of myopia, however, it does not slow down its progression (17, 18). Although the mechanism of action is not yet fully known (19), several theories suggest that dopamine is released during exposure to UV light, thereby reducing the growth of the axial length of the eye (20). Therefore, the risk of developing myopia is 2.6 times higher in children with low exposure to sunlight and high near-vision time (21).

Several studies relate the increase in myopia progression to near-vision work (22, 23). The prolonged use of electronic devices also seems to be related to this increase in the risk of developing myopia among children (24, 25). Smaldone et al. demonstrated in 2015 that children with myopia spent 0.95 h a day in front of a computer, while non-myopic children spent 0.69 h a day (26). Likewise, higher exposure to computers and screens among university students has been associated with an increase in myopia (27). For this reason, in 2016, the American Academy of Optometry recommended limiting screen time in children aged between 2 and 5 to 1 h a day, and they also recommended reducing the use of electronic devices among children over the age of 6 (28). However, this is a controversial topic, and some researches performed between 2009 and 2014 concluded that doing near-vision activities was not a risk factor for the development of myopia (29–31). In these cases, the use of digital devices was not considered. In 2009, Dirani et al. (32), have studied if the screen-time increase is the only risk factor that can be modified to prevent myopia. Facing this question, other authors have written about the importance of considering a historical perspective to propose preventive interventions. These authors proposed preventive interventions to break established behaviors of children, making them spend more time outdoors, as far as there is no scientific evidence about the danger of computers use vs. spending the same time reading (33).

This study reviewed the association of screen time use and outdoor activity time with rates of myopia in Spanish children aged between 5 and 7 influence on their vision.

## Materials and Methods

### Data Collection and Inclusion Criteria

A cross-sectional epidemiological study was conducted in Spain in 2016, 2017, and 2019. The study population was comprised of children aged between 5 and 7 years. The data was collected using the convenience sampling method as part of the “Visual health campaign for schoolchildren,” and it was carried out in optical centers located in the different autonomous communities of Spain. This campaign was aimed at all schools in Spain, meaning therefore that all children aged between 5 and 7 years whose parents would have read and signed the informed consent were included in the study.

The research described herein adhered to the tenets of the Declaration of Helsinki and approved by the ethics investigation committee of Universidad Europea de Madrid (CEI-UE) under the code CIPI/19/102.

To standardize the protocol, which was to be followed, the optometrists who collaborated in the study completed a training course before starting the campaign every year. The course was taught by one optometrist from the vision research group from Universidad Europea. In this course, they were informed of the bases of research, and likewise, the optometric tests included in the protocol were explained. Besides, guidelines were drawn up in which the proposed methodology and possible results were indicated and these were distributed amongst all of the optical centers.

The procedure started with:

A questionnaire that included questions about:

- The demographic data of the children (city of residence, age, sex, and nationality)
- Information about the time spent outside (daylight hours per day classified as high, 2.7+ h, moderate, from 1.6 to 2.7 h or low, from 0 to 1.6 h)
- Information about the time spent doing close vision tasks per day, not including school time. This includes reading, homework, handheld games, drawing, computer work. Parents should choose between low (0 to 2 h), moderate (2 to 3 h) or high (3+ h).
- Information about how many of the time spent doing close vision tasks per day was with electronic devices (<25%, between 25 and 50% or over 50%)
- Past Medical History (main complaint, ocular and medical records of the children, and family ocular and medical records)

### Visual Examination

The procedure followed with an optometric examination in which visual tests were performed on all of the participants to measure their visual acuity, both with and without correction, performing refraction, and assessing binocular vision and accommodation.

### Understanding the Variables

To determine the children's optical prescription, the spherical equivalent (SE) was calculated and the participants were classified according to their SE into hypermetropic, those whose SE was greater than +0.50 diopters; myopic, those whose SE was < -0.50 Diopters or emmetropic, those whose SE was between−0.50 and +0.50, Diopters (34). The formula used for calculating the SE was as follows: SE = (sphere) + (cylinder)/2.

The Clinical Myopia Profile classification was used to calculate the number of hours children spent outdoors, performing near-vision activities, using electronic devices and the influence of their family history (35). According to Gifford, the time the children that spend outdoors during the day (exposure to sunlight) can be classified as low (if they spend between 0 and 1.6 h a day), moderate (if they spend between 1.6 and 2.7 h a day) or high (if they spend more than 2.7 h a day). Concerning near-vision time (excluding school hours), it can be classified as high (more than 3 h a day), moderate (2–3 h a day), or low (0-2 h a day). Within this time interval, the percentage of time spent using digital devices was determined as: <25%, between 25 and 50%, and >50%. With regards to genetics, the questionnaire asked whether either parent or both parents were short-sighted.

### Statistical Analysis

Statistical analysis was conducted using the SPSS 25.0 software (SPSS Inc., Chicago, Illinois). The descriptive analysis of quantitative variables was performed using the mean, standard deviation, confidence interval and odds ratio. The qualitative variables were analyzed using the distribution of frequencies and percentages. Given that they followed a non-parametric distribution on the Kolmogorov-Smirnov test, the Kruskal-Wallis test was used for data analysis. Prevalence was calculated with a 95% confidence interval and likewise, a cut-off point of p ≥0.05 was considered to assess the statistical significance.

## Results

**Figure 1:**
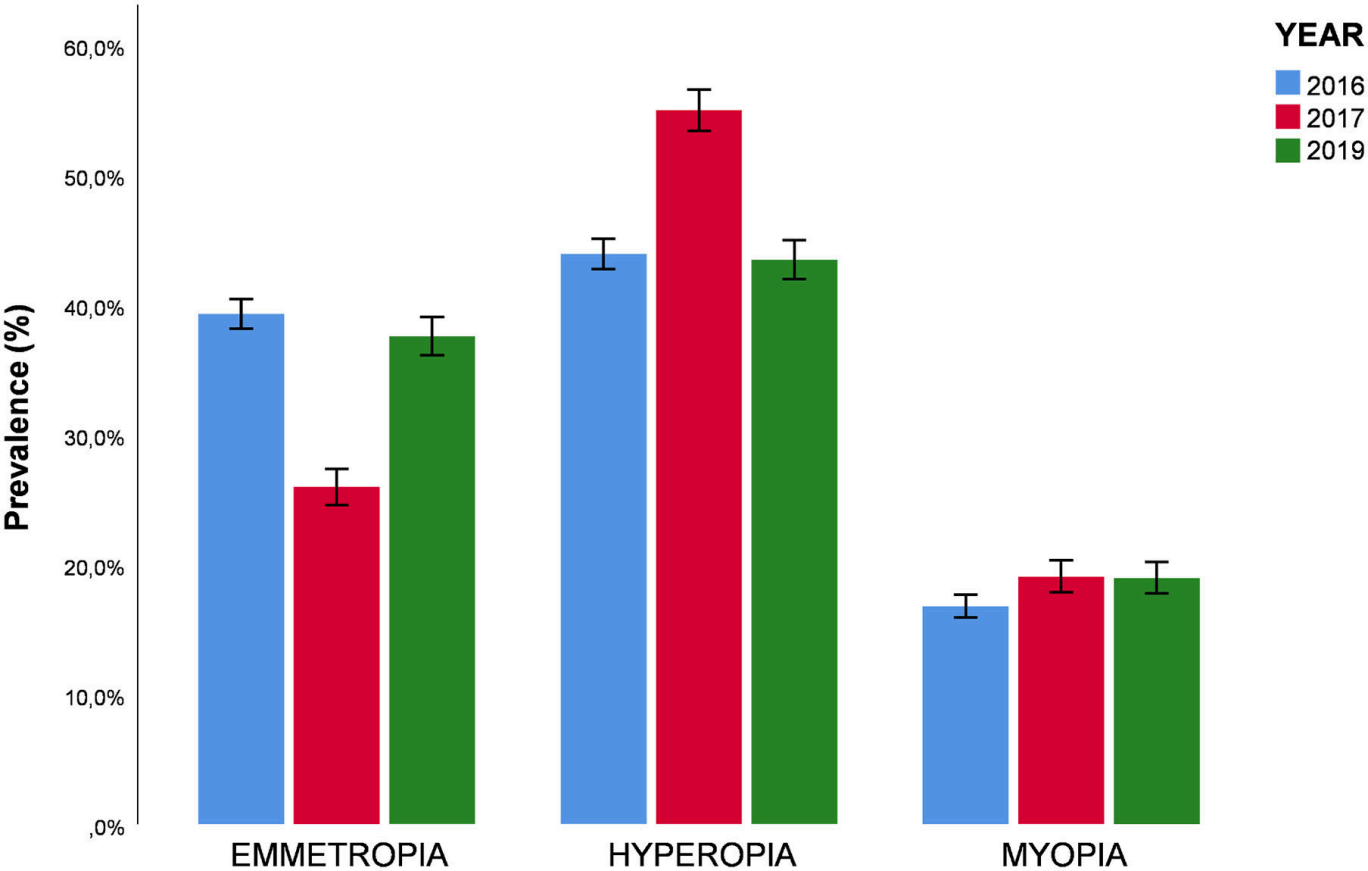
Prevalence of ametropies in 2016, 2017, and 2019.

The study was conducted during the month of September in 2016, 2017 and 2019, coinciding with the beginning of the school year. A total of 8,408 medical records from all over Spain were examined (3,991 in 2016, 2,161 in 2017, and 2,257 in 2019). Of these records, 711 were excluded because they did not meet the inclusion criteria (children were under 5 years or over 7 years of age), and 199 due to missing data. In total, 7,497 participants were examined, of which 35.5% (*n* = 2658) were emmetropic, 46.6% (*n* = 3493) were hypermetropic and 18% (*n* = 1346) were myopic. The percentages vary across the years (Figure 1). The average age of participants was 6.16 ± 0.78 years. In terms of gender, 4,027 (53.7%) of the participants were male, and 3,471 (46.3%) were female.

The mean SE value was 0.80 ± 2.03 Diopters (0.00 ± 0.15D in emmetropia, 2.34 ± 1.67D in hypermetropy, and−1.64 ± 1.54D in myopia). In terms of age, SE at 5 years was 0.97 ± 2.15 Diopters, at 6 years 0.83 ± 2.00 Diopters, and at 7 years 0.66 ± 1.98 Diopters. Regarding gender, mean SE values were 0.81 ± 2.10 Diopters in male participants and 0.78 ± 1.95 Diopters in female participants.

The prevalence of myopic in children aged 5-7 years has increased from 16.8% (*n* = 594) in 2016 to 19% (*n* = 390) in 2019 (OR: 1.14; CI: 1.09−1.19; *p* ≤ 0.001). The prevalence of myopia increased progressively with age (*p* ≤ 0.001) with 13.9% (*n* = 247) in children aged 5 years, 17.6% (*n* = 469) in children aged 6 years and 21.21% (*n* = 630) in children aged 7 years (OR: 6.28; CI: 6.26-6.31; *p* ≤ 0.001). However, no significant association between gender and myopia was detected (*p* = 0.169).

### Near-Vision Activities

The Clinical Myopia Profile classification was used to assess the number of hours that children spent each day doing near-vision tasks. In 2016, 2017, and 2019, 34.7% of children spent more than 3 h a day doing these types of tasks, 25.9% spent between 2 and 3 h a day doing near-vision activities, and 39.4% spent <2 h a day performing said tasks.

Concerning the use of electronic devices, 38.3% of the children who participated in this study spent more than 50% of the said time using them. Only 34.2% spent <25% of the time using them and 27.5% spent between 25 and 50% of the time doing so. From 2016 to 2019 fewer children have near vision activities or excessive screen time of > 3 h and more children have screen time of 1–2 h and the details of the data obtained every year (Table 1). Several programs to improve the lifestyle of children have been established over the last few years; consequently, parents are well aware of the risks that their children face by spending long hours in front of a screen.

**Table 1:** Time spent in near vision and use of digital devices by year.

**Time spent in near vision**		**Low (Between 0 and 2 h /day) *N* (%)**	**Moderate (between 2 and 3 h /day) *N* (%)**	**High (>3 h /day) *N* (%)**
	2016	2,187 (27.6%)	1,198 (23.5%)	2,821 (51.8%)
	2017	1,377 (17.4%)	914 (17.9%)	1,508 (27.7%)
	2019	4,350 (55.0%)	2,992 (58.6%)	1,114 (20.5%)
**Use of electronic devices**		** <25%** ***N*** **(%)**	**Between 25 and 50%** ***N*** **(%)**	**>50%** ***N*** **(%)**
	2016	1,330 (18.6%)	1,236 (22.8%)	3,640 (61.7%)
	2017	1,251 (17.5%)	1,292 (23.8%)	1,256 (21.3%)
	2019	4,560 (63.9%)	2,890 (53.3%)	1,006 (17.0%)

The number of hours spent in near vision and the use of electronic devices increase significantly with age, with this number being higher in children aged 7 years (OR:1.02; CI:0.99-1.94; *p* < 0.05). Thus, older children are spending more time on devices, especially those with excessive screen time > 3 h (Tables 2, 3). The increasingly frequent use of digital devices, both at home and at school, means that children are becoming more and more dependent on them as they grow older.

**Table 2:** Odds ratio and confidence Interval in outdoor activities, near activities and the use of digital devices depending on age and refractive error.

		**5 years OR (IC)**	**6 year OR (IC)**	**7 years OR (IC)**	**Total OR (IC)**
**Outdoor activities**	Emmetropia	0.84 (0.80–0.88)	0.82 (0,78–0.86)	0.83 (0.79–0.86)	0.83 (0.81–0.85)
	Hyperopia	0.76 (0.73–0.80)	0.76 (0.73–0.79)	0.71 (0.68-0.74)	0.74 (0.72–0.76)
	Myopia	0.76 (0.70–0.82)	0.75 (0.71–0.80)	0.78 (0.74–0.82)	0.77 (0.74–0.79)
**Use of electronic devices**	Emmetropia	0.94 (0.85–1.05)	1.08 (0.97–1.20)	1.01 (0.91–1.12)	0.96 (0.90–1.02)
	Hyperopia	0.87 (0.78–0.96)	1.13 (1.02–1.24)	0.86 (0.78–0.94)	0.90 (0.85–0.95)
	Myopia	0.66 (0.59–0.73)	0.92 (0.81–1.05)	1.28 (1.13–1.44)	1.13 (1.04–1.21)
**Near activities**	Emmetropia	1.11 (0.98–1.25)	1.00 (0.90–1.10)	1.11 (1.01–1.23)	1.04 (0.98–1.10)
	Hyperopia	0.98 (0.87–1.01)	1.00 (0.90–1.09)	1.07 (0.97–1.18)	0.98 (0.92–1.04)
	Myopia	1.25 (1.06–1.47)	1.01 (0.89–1.15)	1.02 (0.91–1.14)	1.09 (1.01–1.18)

**Table 3:** Frequency distribution of time spent in near vision, using digital devices and in outdoor activities depending on the children's age.

		**Low (between 0 and 2 h/day)**	**Moderate (between 2 and 3 h/day)**	**High (>3 h/day)**
**Time spent in near vision**	5 years	1123 (28.4%)	654 (25.6%)	534 (19.6%)
	6 years	1421 (35.9%)	916 (35.9%)	984 (36.2%)
	7 years	1410 (35.7%)	982 (38.5%)	1203 (44.2%)
	Total	3954 (100.0%)	2552 (100.0%)	2721 (100.0%)
**Use of electronic devices**		** <25%**	**between 25 and 50%**	**>** **50%**
	5 years	1005 (28.1%)	657 (24.3%)	651 (22.1%)
	6 years	1251 (35.1%)	979 (36.2%)	1090 (36.9%)
	7 years	1313 (36.8%)	1072 (39.6%)	1210 (41.0%)
	Total	3569 (100.0%)	2708 (100.0%)	2951 (100.0%)
**Outdoor activities**		**Low (between 0 and 1.6 h/day)**	**Moderate (between 1.6 and 2.7 h/day)**	**High (>2.7 h/day)**
	5 years	627 (25.1%)	695 (27.2%)	290 (26.2%)
	6 years	876 (35.1%)	874 (34.2%)	399 (36.1%)
	7 years	991 (39.7%)	990 (38.7%)	417 (37.7%)
	Total	2494 (100.0%)	2559 (100.0%)	1106 (100.0%)

About the spherical equivalent value, the more time spent in near-vision activities and using electronic devices, the more significant the trend toward myopization (Table 4). Furthermore, significant differences have been observed when comparing the number of hours spent in near vision activities in the different autonomous communities of Spain (*p* ≤ 0.001).

**Table 4:** Spherical equivalent according to the time spent in near-vision activities and the use of electronic devices.

		**Low (between 0 and 2 h/day)**	**Moderate (between 2 and 3 h/day)**	**High (>3 h/day)**	**Total**
**Time spent in near vision**	5 years	1.02 ± 2.22	0.98 ± 2.14	0.95 ± 2.15	0.99 ± 2.18
	6 years	0.83 ± 1.99	0.82 ± 2.07	0.84 ± 1.94	0.83 ± 1.99
	7 years	0.80 ± 1.99	0.65 ± 1.91	0.64 ± 1.97	0.69 ± 1.96
**Use of electronic devices**		** <25%**	**Between 25 and 50%**	**>50%**	**Total**
	5 years	1.00 ± 2.25	1.08 ± 2.12	0.90 ± 2.13	0.99 ± 2.18
	6 years	0.86 ± 2.09	0.87 ± 1.93	0.78 ± 1.95	0.83 ± 1.99
	7 years	0.85 ± 1.88	0.68 ± 2.02	0.59 ± 1.98	0.79 ± 1.96

**Figure 2:**
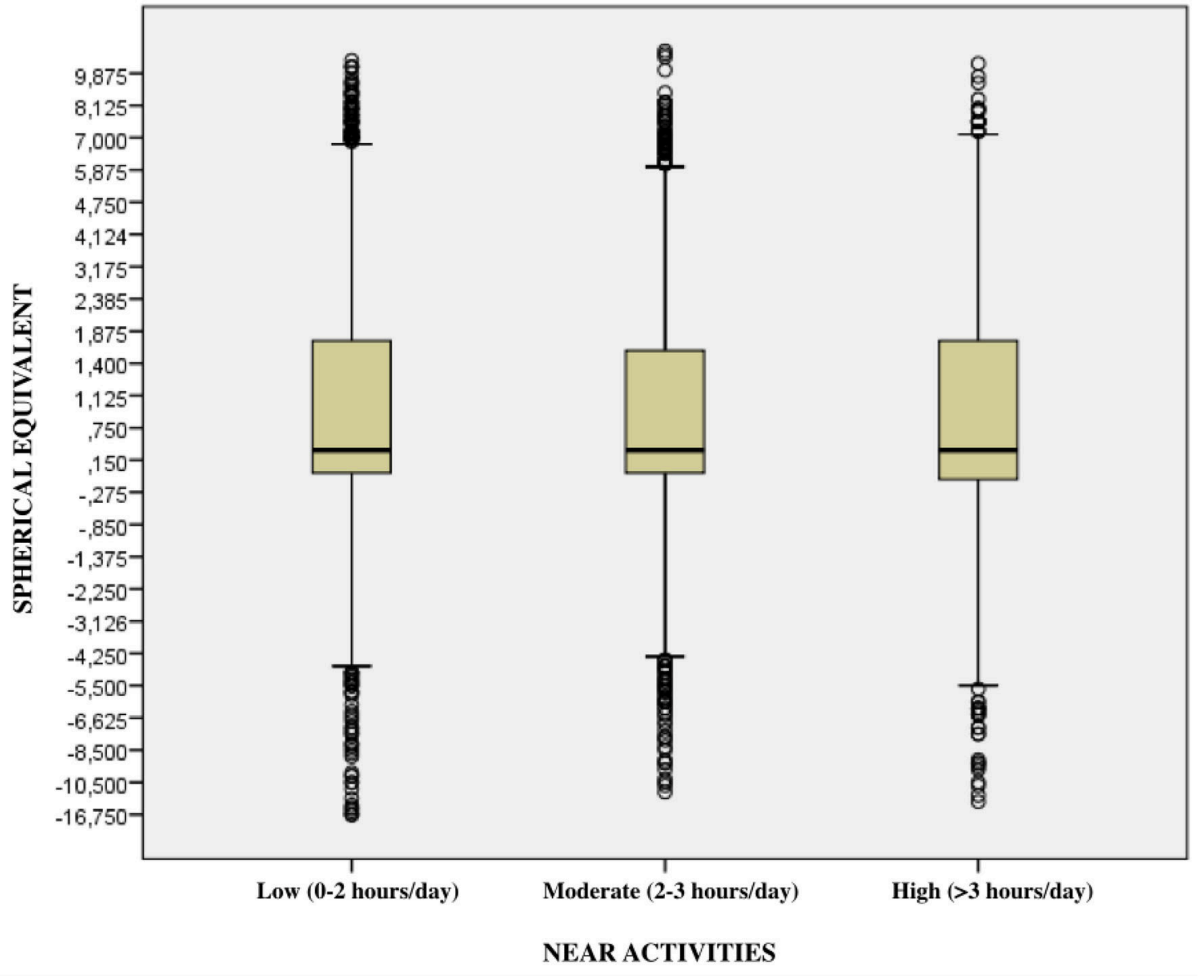
Relationship between near work and the magnitude of refractive error (EE).

**Figure 3:**
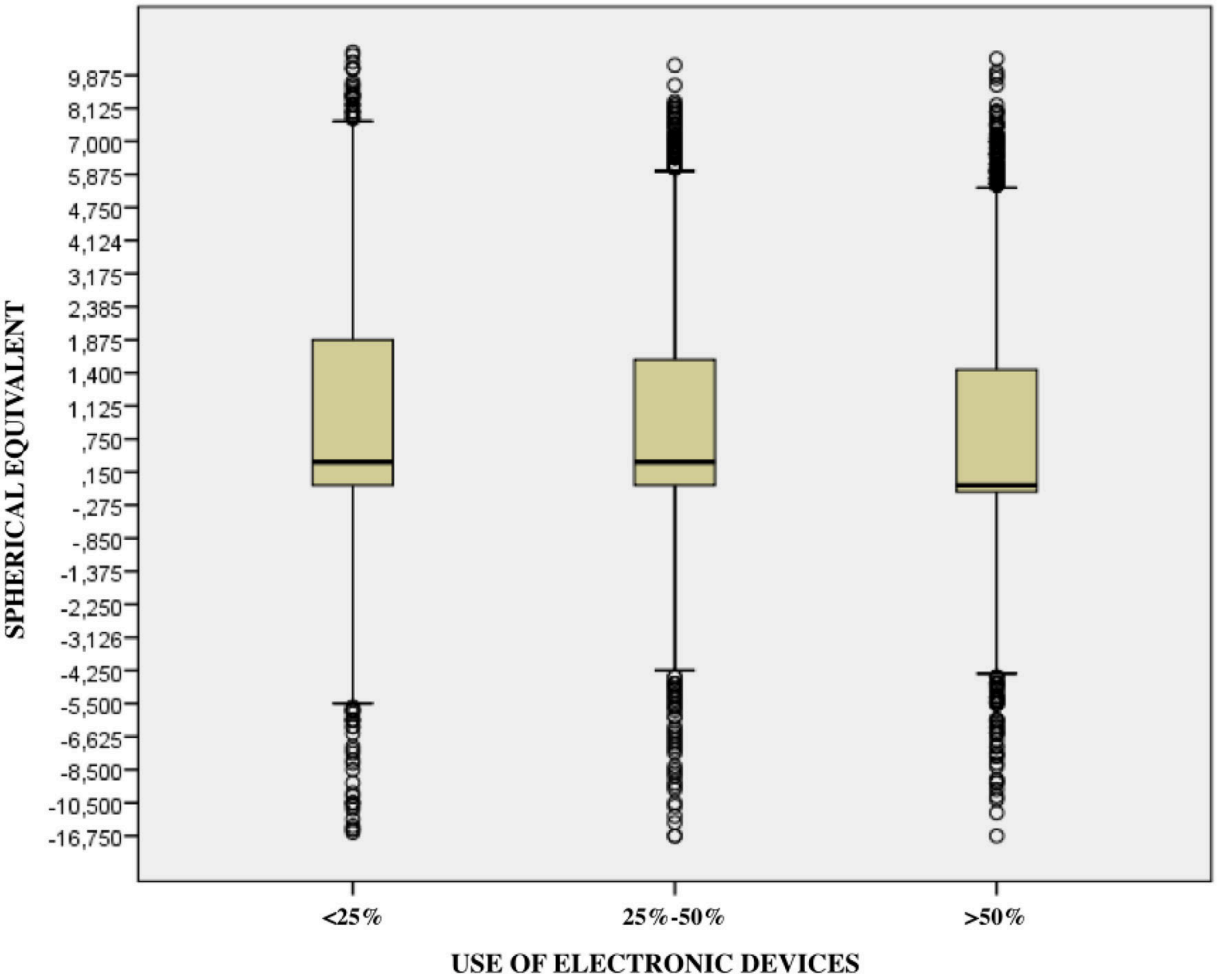
Relationship between the use of digital devices and the magnitude of refractive error.

Therefore, there is a clear association between the excessive use of electronic devices and the increased prevalence of myopia (OR: 1.10; CI: 1.07-1.13; *p* ≤ 0.001) (Figures 2, 3).

### Outdoor Activities

**Figure 4:**
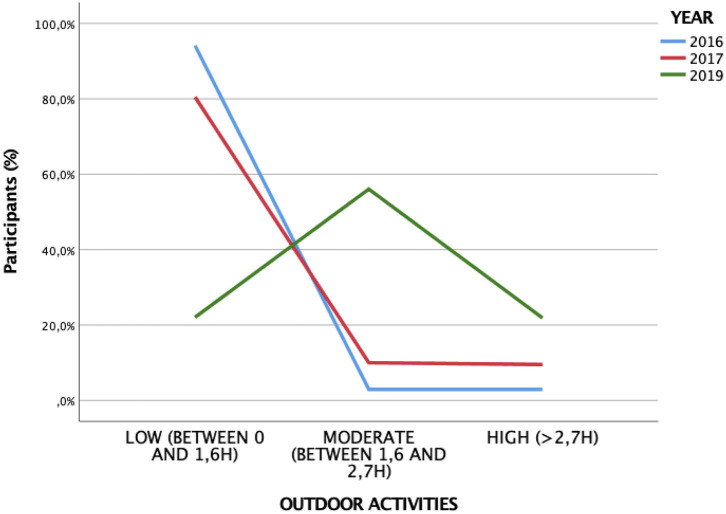
Outdoors activities by age.

**Figure 5:**
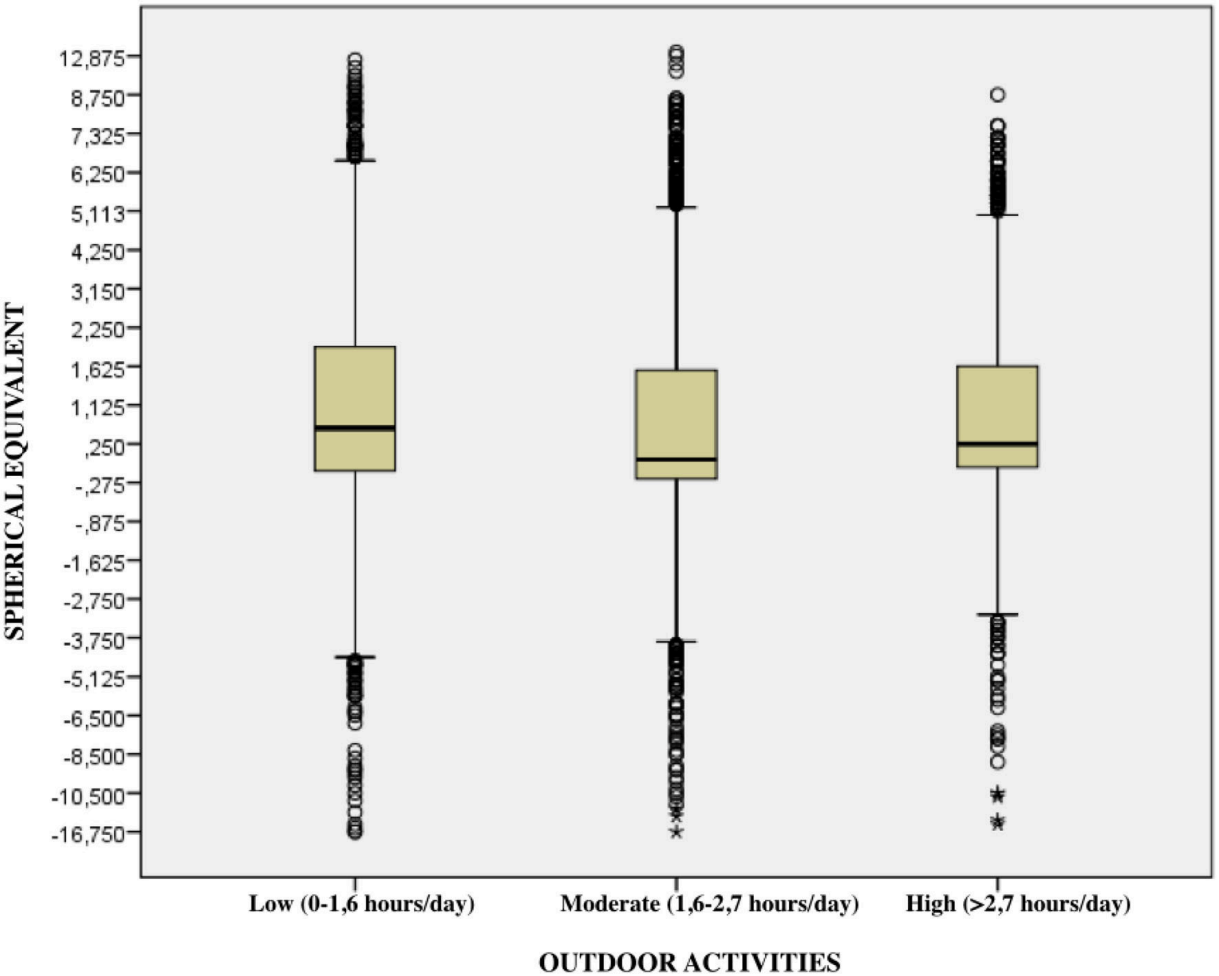
Relationship between hours in outdoor activities and the magnitude of refractive error.

Based on the Clinical Myopia Profile classification, 50.6% of the children who participated in the study spent between 0 and 1.6 h exposed to UV light each day, 33.2% between 1.6 and 2.7 h a day, and only 16.2% spent more than 3 h a day outdoors (Table 2, Figure 4). Myopia decreases as the time spent exposed to the UV light increases can be observed (*p* ≤ 0.001) (Figure 5). Moreover, the number of hours that children spend outdoors decreases with age (Table 3) (*p* ≤ 0.001).

## Discussion and Conclusions

In this study, we had three main findings. Firstly, the increased time spent on near activities and using digital devices was associated with higher rates of myopia in Spanish children. Secondly, prevalence rates of myopia in children aged 5–7 years are increasing. Thirdly children who were reported to spend more time outdoors were less likely to develop myopia

Regarding the first finding, we have got that the time spent doing near activities has a direct impact on the prevalence of myopia in children aged between 5 and 7 (OR>1). Nevertheless, when we checked the time of these near activities that children spend with digital devices, we have got that the percentage of 7 years old children that spent more than 50% of the time doing near activities with electronic devices is higher than in children of 5 and 6 years old. Our study shows that, in general, the more the time using digital devices the higher the myopia prevalence (OR>1). But it is important to point out that we have found differences with age, meaning that we have not got a relation in the use of digital devices and myopia in children of 5 and 6 years old, but there is a relationship in children of 7 years old. Multiple studies also sustain that excessive use of smartphones, computers, television, etcetera, as well as the hours spent doing near-vision activities, have a negative impact on vision, and increase the risk of developing problems (15, 26, 31). For instance, the prevalence of myopia in children from Sydney (*n* = 124) was compared to the same in children from Singapore (*n* = 628), finding that it was higher in Singapore (29.1 vs. 3.3%) as a result of the differences in the children's lifestyles of both countries, considering that in Singapore they spent more hours reading books and doing near activities while in Sydney spent more time in outdoor activities (13,75 vs. 3,05 h a week in Singapore) (21). Other researchers have related a higher risk of developing myopia with shorter distances for reading (<20 cm) and longer and continuous periods (>45 min), instead of joining to the total time in near activities (17). In this sense, a recent study has concluded that results are mixed and that more studies are needed to evaluate the association between screen time and myopia (36).

About the second main finding, the prevalence of myopia in Spanish children aged 5–7 years was 19%. This number is similar to the prevalence of myopia in children in other countries as Australia, with a 14.02% in children of primary school in 2005 (37), Central Asia or Latin America, with 17 and 20.5% respectively (38). When compare to other countries in Europe, we also find that prevalence of myopia increase with age, as in England, with a 9.4% of prevalence in children aged 6–7 years that increased to 29.4% in children aged 12–14 years (39). Comparing to Africa, myopia rates are lower in Nigeria, probably due to less access to digital devices for children (40, 41).

Finally, our third main finding was the association between more time spent outdoors and lower rates of myopia. Ho et al. recommended, after reviewing different studies, that children spend their break times outdoors and that they spend an additional hour outdoors after school every day to prevent myopia (42). Although the mechanism of action is still unknown, recent researches have considered several theories, as the protective effect of blue light or the increase in the blood of D-vitamin due to UV exposure. Others support the hypothesis of dopamine is one of the retinal neurotransmitters involved in the growth of the axial length. So, the protective effect of outdoor activities is partly due to the stimulation of light in the release of dopamine in the retina (43).

Children today are born into the digital age and are immersed in new technologies. Thus, computers, videogames, smartphones, tablets, and the internet are an essential part of their lives and have become their technological reality (44). Lifestyles have changed drastically as a result of the emergence of these new technologies, which have brought numerous benefits but also some risks that must be analyzed to minimalize them. Thereby, by knowing the risks that the use of digital devices can have on vision, it is possible to reduce these risks, by proposing preventive strategies that help to ensure the normal development of visual capacities.

Our study had several limitations. We had fewer numbers of younger children. Screen time was an estimate based on recall and not objectively measured. Similarly, outdoor-time was not objectively measured. It is also important to note that the campaign offered free glasses to the children that needed them, which could be considered a bias for this study.

In conclusion, this research confirms that excessive use of electronic devices and lower exposure to the outdoors causes a higher risk for children aged between 5- and 7-years developing myopia.

## Data Availability Statement

The raw data supporting the conclusions of this article will be made available by the authors, without undue reservation.

## Ethics Statement

The studies involving human participants were reviewed and approved by Comité de Ética de la Investigación de la Universidad Europea. Written informed consent to participate in this study was provided by the participants' legal guardian/next of kin.

## Author Contributions

All authors contributed in the conceptualization, methodology, analysis, writing, and approved the final version of the manuscript.

## Conflict of Interest

The authors declare that the research was conducted in the absence of any commercial or financial relationships that could be construed as a potential conflict of interest.
